# Intramuscularly Administered Enterotoxigenic *Escherichia coli* (ETEC) Vaccine Candidate MecVax Prevented H10407 Intestinal Colonization in an Adult Rabbit Colonization Model

**DOI:** 10.1128/spectrum.01473-22

**Published:** 2022-06-28

**Authors:** Ipshita Upadhyay, Kathryn L. Lauder, Siqi Li, Galen Ptacek, Weiping Zhang

**Affiliations:** a University of Illinois at Urbana-Champaign, Department of Pathobiology, Urbana, Illinois, USA; University of Guelph

**Keywords:** MecVax, ETEC (enterotoxigenic *Escherichia coli*), intestinal colonization, rabbit model, vaccine, diarrhea

## Abstract

Currently, there are no vaccines licensed for enterotoxigenic Escherichia coli (ETEC), a leading cause of children’s diarrhea in developing countries and the most common cause of travelers’ diarrhea. A vaccine preventing ETEC bacteria from colonization at small intestines and neutralizing enterotoxin toxicity is expected to be effective against ETEC diarrhea. Protein-based multivalent vaccine candidate MecVax was demonstrated recently to induce antibodies neutralizing heat-labile toxin (LT) and heat-stable toxin (STa) enterotoxicity and inhibiting adherence of seven ETEC adhesins (CFA/I, CS1 to CS6) but also to protect against ETEC toxin-mediated clinical diarrhea in a pig challenge model. To further evaluate MecVax preclinical efficacy against ETEC colonization at small intestines, in this study, we intramuscularly immunized adult rabbits with MecVax, challenged rabbits with ETEC strain H10407 (CFA/I, LT, STa), and examined prevention of bacteria intestinal colonization. Data showed that rabbits immunized with MecVax developed antibodies to both ETEC toxins (LT, STa) and seven adhesins (CFA/I, CS1 to CS6) and had over 99.9% reduction of H10407 intestinal colonization, indicating that the broadly immunogenic ETEC vaccine candidate MecVax is protective against ETEC H10407 intestinal colonization. This study also confirmed that parenteral administration of a protein-based vaccine can prevent bacteria intestinal colonization. Protection against ETEC intestinal colonization demonstrated by this rabbit study, in conjugation with protection against ETEC enterotoxin-mediated clinical diarrhea from a previous pig challenge study, suggested that MecVax can potentially be an effective ETEC vaccine and a combined pig and rabbit challenge model can evaluate ETEC vaccine preclinical efficacy.

**IMPORTANCE** An effective ETEC vaccine would prevent hundreds of millions of diarrhea clinical cases and save nearly 100,000 lives annually. MecVax, a protein-based injectable multivalent ETEC vaccine candidate, has been shown for the first time to induce functional antibodies against both ETEC enterotoxins (STa, LT) produced by all ETEC strains and seven ETEC adhesins (CFA/I, CS1 to CS6) expressed by ETEC strains causing a majority of ETEC diarrhea clinical cases and the moderate-to-severe cases. Moreover, MecVax was demonstrated to protect against ETEC STa or LT toxin-mediated diarrhea in a pig model. If it also protects against ETEC intestinal colonization, MecVax can be validated as an effective ETEC vaccine candidate. This adult rabbit colonization model study showed that intramuscular administration of MecVax effectively prevented intestinal colonization by H10407, perhaps the most virulent ETEC strain, affirming MecVax vaccine candidacy and accelerating vaccine development against ETEC children’s diarrhea and travelers’ diarrhea.

## INTRODUCTION

Enterotoxigenic Escherichia coli (ETEC) strains expressing enterotoxins, heat-labile toxin (LT) and heat-stable toxin (STa), and immunologically heterogeneous fimbrial or nonfimbrial adhesins, colonization factor antigens (CFAs) and coli surface antigens (CSs), are a leading cause of children’s diarrhea in developing countries (1–4) and the most common cause of travelers’ diarrhea (5–7). Currently, there are no effective control measures or licensed vaccines to protect children and international travelers from ETEC-associated diarrhea (8).

A vaccine providing broad protection against enterotoxicity of ETEC enterotoxins LT and, especially, STa and also against adhesin-mediated bacterial adherence to host receptors and colonization at small intestines would be effective against ETEC children’s diarrhea and travelers’ diarrhea (9, 10). ETEC vaccine candidates currently under clinical investigation primarily target ETEC adhesin antigens and LT toxin (11–15), but they do not carry STa toxin antigens to induce protective antibodies against this key ETEC toxin, which plays a more important role in causing children’s diarrhea and travelers’ diarrhea (1, 2, 16). Different from those candidates, MecVax, an injectable multivalent vaccine candidate composed of two polyvalent protein immunogens, ETEC toxoid fusion 3xSTa_N12S_-mnLT_R192G/L211A_ and adhesin multiepitope fusion antigen (MEFA) CFA/I/II/IV, induces functional antibodies against adherence of the seven most important ETEC adhesins (CFA/I, CS1 to CS6) and enterotoxicity of LT but also STa toxins (17–19). Toxoid fusion protein 3xSTa_N12S_-mnLT_R192G/L211A_, which has three copies of STa toxoid STa_N12S_ genetically fused to double mutant LT toxoid monomer mnLT_R192G/L211A_ (a double mutant LT A subunit fused to an LT B subunit as a single polypeptide) (20), induces antitoxin antibodies neutralizing enterotoxicity of both ETEC toxins and, more importantly, protects against STa- or LT-mediated clinical diarrhea in a pig challenge model (17, 20–22). ETEC adhesin CFA/I/II/IV MEFA protein, an epitope- and structure-based multiepitope fusion antigen protein (23), presents functional epitopes of seven ETEC adhesins, CFA/I, CS1, CS2, CS3, CS4, CS5, and CS6, on CFA/I adhesin major subunit CfaB backbone (24). This CFA/I/II/IV MEFA protein induces broad anti-adhesin antibodies that inhibit adherence of E. coli or ETEC strains expressing any of the seven target adhesins (CFA/I, CS1 to CS6) and significantly reduces ETEC strain B7A (CS6, STa, LT) colonization at rabbit small intestines (24–26).

MecVax, a multivalent ETEC vaccine candidate derived from the combination of proteins toxoid fusion 3xSTa_N12S_-mnLT_R192G/L211A_ and CFA/I/II/IV MEFA, was demonstrated recently to protect against ETEC toxin-mediated diarrhea in a pig challenge model. However, preclinical protection from this vaccine candidate against ETEC bacteria intestinal colonization is not determined. In this study, we intramuscularly immunized adult rabbits with MecVax to confirm vaccine candidate broad immunogenicity, and we then orally challenged the immunized rabbits with an ETEC wild-type strain to examine MecVax *in vivo* protection against bacteria intestinal colonization, an adult rabbit colonization model to evaluate MecVax candidacy as an effective ETEC vaccine. Additionally, we intramuscularly immunized a group of rabbits with protein CFA/I/II/IV MEFA, the MecVax adhesin antigen component, to comparatively examine vaccine antigen compatibility, and further explored potential application of parenteral immunization of protein-based vaccines for protection against enteric infection.

## RESULTS

### Intramuscularly administered MecVax elicited robust IgG responses to the targeted virulence factors in adult rabbits.

**FIG 1:**
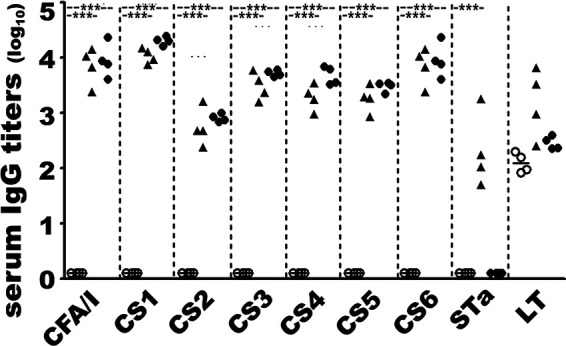
Rabbit serum IgG antibody titers (log_10_) from the immunized and control groups. Serum samples from individual rabbits (four rabbits per group) treated intramuscularly with MecVax (▴), adhesin CFA/I/II/IV MEFA protein (●), or PBS (○), adjuvanted with dmLT, were titrated in ELISAs with heat-extracted CFA/I, CS1, CS2, CS3, CS4, or CS5 adhesin, recombinant protein CS6 subunit CssA, CT (LT homologues), or STa-ovalbumin conjugates as the coat antigen for IgG responses to the seven target adhesins (CFA/I, CS1 to CS6) and LT and STa toxin. Significant differences were determined using one-way ANOVA with Tukey’s test; ***, *P* < 0.001.

Adult rabbits intramuscularly immunized with MecVax, adjuvanted with double mutant LT (dmLT; holotoxin-structured LT_R192G/L211A_), showed no apparent adverse effects and developed robust IgG antibody responses to the adhesins and enterotoxins targeted by the vaccine candidate (Fig. 1). Rabbit serum anti-CFA/I, -CS1, -CS2, -CS3, -CS4, -CS5, -CS6, -STa, and -LT IgG titers were 3.8 ± 0.34, 4.0 ± 0.13, 2.7 ± 0.34, 3.5 ± 0.25, 3.3 ± 0.23, 3.3 ± 0.24, 3.8 ± 0.34, 2.3 ± 0.67, and 3.2 ± 0.62 (log_10_), respectively.

Rabbits intramuscularly immunized with the adhesin antigen component of MecVax, CFA/I/II/IV MEFA protein (without the toxoid fusion protein), adjuvanted with dmLT, developed the same levels of anti-adhesin IgG (Fig. 1). Anti-CFA/I, -CS1, -CS2, -CS3, -CS4, -CS5, and -CS6 serum IgG titers were 3.9 ± 0.32, 4.3 ± 0.07, 2.9 ± 0.07, 3.7 ± 0.05, 3.7 ± 0.16, 3.5 ± 0.09, and 3.9 ± 0.32 (log_10_). While rabbits immunized with adhesin CFA/I/II/IV MEFA protein developed no response to STa toxin, they showed anti-LT IgG titers of 2.4 ± 0.12 (derived from the dmLT adjuvant). No antigen-specific IgG responses except anti-LT IgG antibodies (2.1 ± 0.18; log_10_), which were derived from adjuvant dmLT, were detected from serum samples of the control rabbits (intramuscularly administered with phosphate-buffered saline [PBS] and adjuvant dmLT).

IgG and IgA specific to the toxins or adhesins targeted by MecVax were recorded not detectable from rabbit fecal or cecum content supernatants. The enzyme-linked immunosorbent assay (ELISA) optical density (OD) values in the wells with samples from the rabbits immunized with MecVax were raised but were below the OD threshold reading (OD = 0.3) and thus recorded as no antibody detected.

### MecVax-induced rabbit serum antibodies inhibited adherence of E. coli or ETEC bacteria expressing any of the seven target adhesins *in vitro*.

**FIG 2:**
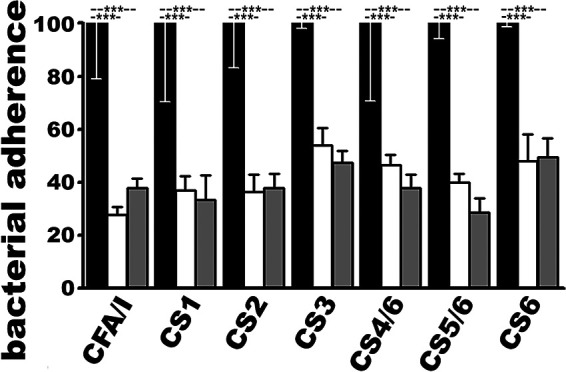
Rabbit serum antibody *in vitro* adherence inhibition against E. coli or ETEC bacteria expressing CFA/I, CS1, CS2, CS3, CS4/CS6, CS5/CS6, or CS6 adhesin. Adherence to Caco-2 cells from H10407 (CFA/I, STa, LT), THK38/pEU405 (CS1), DH5α/pEU588 (CS2), E116 (CS3, STa, LT), E106 (CS4, CS6, STa, LT), UM75688 (CS5, CS6, STa, LT), or 2423 ETP98066 (CS6, STa, LT) bacteria (CFU in %; data point N = 20) after incubation with rabbit serum samples from the group intramuscularly immunized with MecVax (boxes in white) or CFA/I/II/IV MEFA protein (boxes in gray), compared to bacteria adherence after treatment with the serum of the control rabbits (CFU referred to as 100%; boxes in black). Boxes and bars are means and standard deviations. Significant differences were determined using one-way ANOVA with Tukey’s test; ***, *P* < 0.001.

Adherence of E. coli recombinant strains expressing CS1 (THK38/pEU405) or CS2 adhesin (DH5α/pEU588) and ETEC field isolates producing CFA/I (H10407), CS3 (E116), CS4/CS6 (E106), CS5/CS6 (UM75688), or CS6 adhesin (2423 ETP98066) to Caco-2 cells was significantly reduced after bacteria were incubated with serum samples of the rabbits intramuscularly immunized with MecVax (Fig. 2). Adherence from these strains to Caco-2 cells was reduced by 63 ± 5.4, 63.5 ± 6.5, 72 ± 3.0, 46 ± 6.6, 53.5 ± 3.9, 60 ± 3.1, and 52 ± 10.2 (%), respectively, compared to that after bacteria incubation with the control rabbit serum samples.

Adherence to Caco-2 cells from the same bacteria expressing CS1, CS2, CFA/I, CS3, CS4/CS6, CS5/CS6, or CS6 adhesin after incubation with the serum of the rabbit immunized with CFA/I/II/IV MEFA protein was reduced by 66.5 ± 9.0, 62 ± 5.6, 62 ± 3.8, 52.5 ± 4.4, 62 ± 4.9, 71 ± 5.2, and 50.5 ± 7.0 (%), reductions equal to those of cells incubated with the rabbit sera from the group immunized with MecVax.

### Intramuscular administration of MecVax significantly prevented ETEC H10407 from colonization at rabbit small intestines.

**FIG 3:**
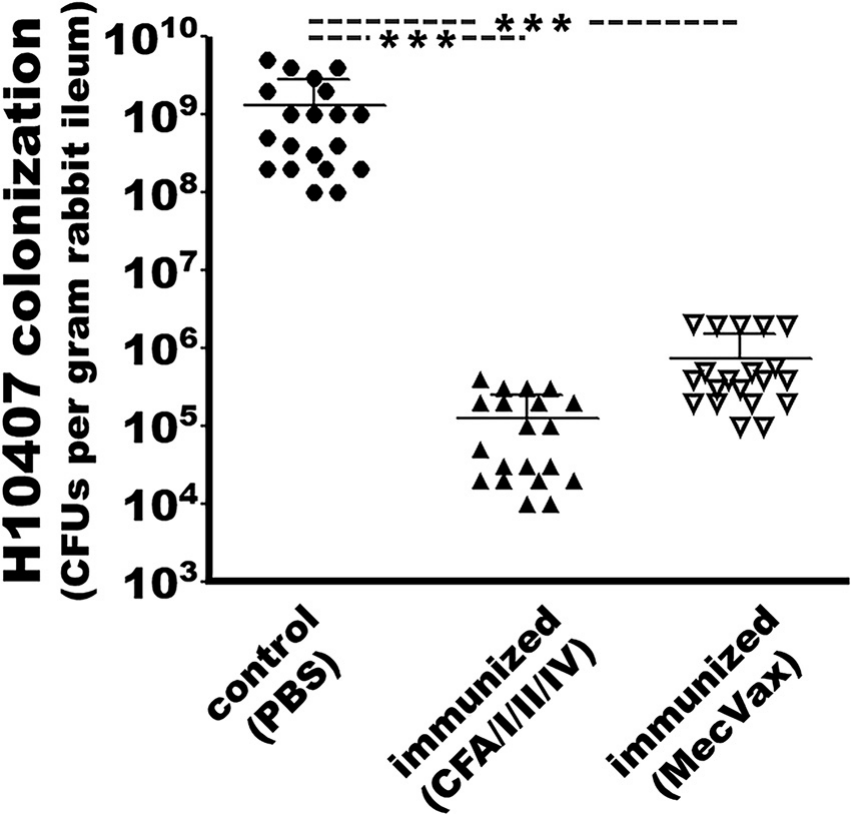
ETEC H10407 (CFA/I, STa, LT) bacterial colonization at small intestines of the immunized or control rabbits. Distal ileal segment was collected from each rabbit (four rabbits per group; five data points per rabbit, *n* = 20) which was intramuscularly administered with MecVax, CFA/I/II/IV MEFA protein, or PBS (adjuvanted with dmLT) and orogastrically challenged with ETEC H10407, rinsed with PBS (to remove fecal content), ground, serially diluted, and plated on MacConkey agar plates. After grown overnight at 37°C, bacteria (in CFU) were counted and recorded. Bars are means and standard deviations. Significant differences were determined using one-way ANOVA with Tukey’s test; ***, *P* < 0.001.

After orogastric inoculation with ETEC strain H10407, none of the rabbits showed diarrhea, loose feces, fluid accumulation in small intestines, any visually apparent intestinal damage, or other clinical symptoms. However, those intramuscularly immunized with MecVax had over 99.9% reduction of bacterial colonization at small intestines compared to that of the control rabbits (*P* < 0.001) (Fig. 3). The number of bacteria (CFU) per gram ileal tissue of the rabbits immunized with MecVax was 7.5 ± 7.7 (×10^5^), whereas the number of bacteria (CFU) from the control rabbit ileal tissue was 1.3 ± 1.5 (×10^9^).

Rabbits intramuscularly immunized with MecVax adhesin antigen component CFA/I/II/IV MEFA also showed almost 4-log reduction of H10407 colonization at small intestines. Number of bacteria (CFU) per gram ileal tissue was 1.3 × 10^5^ ± 1.2 × 10^5^, which was significantly lower than the number of bacteria from the control rabbit ileal tissue (*P* < 0.001) but not statistically different from the number of bacteria from the ileum of the rabbits immunized with MecVax.

Colonies randomly selected were 100% positive when they were tested in PCR with primers specific to CFA/I major subunit CfaB, indicating that the bacteria collected from rabbit ilium segments were the challenge ETEC strain.

## DISCUSSION

Because ETEC strains expressing CFA/I, CS1, CS2, CS3, CS4, CS5, or CS6 adhesin and STa and/or LT enterotoxin are responsible for over 60% of ETEC diarrhea clinical cases and the moderate-to-severe cases (27–30), these seven adhesins and two toxins become the primary antigen targets in ETEC vaccine development. An ETEC vaccine inducing protective immunity inhibiting adherence from these seven adhesins and neutralizing enterotoxicity of LT and STa enterotoxins is expected to be protective against about two thirds of ETEC diarrhea clinical cases. However, the ETEC vaccine candidates currently under clinical safety and immunogenicity or efficacy studies cover several adhesins and LT toxin, but none of them carry antigens of all the seven adhesins and particularly STa toxin. The 19-amino-acid potent STa toxin is poorly immunogenic; exposure to STa toxin does not mount anti-STa antibodies and does not confer protection against STa ETEC infection. Because STa toxin plays a more important role in causing ETEC-associated children’s diarrhea and travelers’ diarrhea, ETEC vaccines without providing protection against this key toxin are unlikely to be effective against ETEC.

MecVax, a multivalent ETEC vaccine candidate carrying polyvalent proteins LT-STa toxoid fusion and CFA/I/II/IV MEFA, was demonstrated to induce functional antibodies inhibiting adherence of seven adhesins (CFA/I, CS1 to CS6) and neutralizing enterotoxicity of LT and especially STa toxin (17–19). Moreover, MecVax was shown to induce IgG and IgA antibodies to the target adhesins and toxins in pregnant pigs after intramuscular immunization, and vaccine-derived passive antibodies protected the newly born piglets from clinical diarrhea after oral inoculation with an STa- or an LT-positive ETEC recombinant strain (producing STs or LT toxin and an adhesin specific to pigs) (18). While LT and STa toxin expressed by ETEC strains causing diarrhea in humans and pigs are highly homologous (31), ETEC adhesins are specific to pigs and humans since two species likely have different host receptors. Thus, ETEC strains expressing pig-type adhesins colonize pig small intestines, and strains with human-type adhesins colonize only human small intestines. Therefore, while a pig challenge model allowed us to evaluate MecVax preclinical efficacy against clinical diarrhea mediated by ETEC STa or LT toxin, the pig model was not able to assess efficacy of MecVax against ETEC bacteria colonization at small intestines.

Rabbits, on the other hand, can be colonized by human ETEC strains when a high oral dose and restriction on fluid movement or peristalsis are applied (32–34). Indeed, we recently demonstrated that adult rabbit small intestine can be colonized by B7A, an ETEC wild-type strain expressing CS6 adhesin and LT and STa toxins; more interestingly, that study showed that rabbits intramuscularly immunized with CFA/I/II/IV MEFA protein, the adhesin antigen component of MecVax, had 2-log reduction of B7A bacteria intestinal colonization (26). However, the limitation of this model is that adult rabbits typically do not develop diarrhea or show other clinical symptoms after ETEC infection and are not susceptible to STa toxin. Therefore, the adult rabbit colonization model, while it can assess protection against ETEC intestinal colonization, is not applicable to evaluate vaccine preclinical efficacy against ETEC clinical diarrhea. Additionally, that early study examined protection from CFA/I/II/IV MEFA protein but not MecVax against ETEC bacteria intestinal colonization (26). By testing CFA/I/II/IV MEFA protein after combining it with the toxin antigen component of MecVax, toxoid fusion protein 3xSTa_N12S_-mnLT_R192G/L211A_, in the rabbit colonization model, we can determine if this adhesin antigen in the vaccine product remains fully protective and whether MecVax is effective against ETEC intestinal colonization.

A combination of a pig challenge model to evaluate efficacy against ETEC toxin-mediated diarrhea and a rabbit colonization model to assess protection against ETEC bacteria intestinal colonization results in a dual animal challenge model, and this dual challenge model allows us to evaluate MecVax vaccine candidacy against ETEC infection. Results from the current rabbit model study revealed that MecVax protects against ETEC H10407 colonization at small intestines, shown by an over 99.9% reduction of intestinal colonization by the challenged ETEC strain in the rabbits immunized with MecVax. In conjunction with the results from a previous study in which MecVax protected against STa- or LT-mediated ETEC diarrhea (18), MecVax unprecedentedly protects against ETEC intestinal colonization and ETEC toxin-mediated diarrhea. Therefore, MecVax becomes the first ETEC vaccine candidate protecting against ETEC toxin enterotoxicity, adherence, and intestinal colonization and thus is potentially effective against ETEC children’s diarrhea and travelers’ diarrhea. Data from this study also showed that rabbits immunized with MecVax or its adhesin antigen CFA/I/II/IV were equally protected from H10407 intestinal colonization, confirming that the two antigen components of MecVax are fully compatible antigenically. Since CFA/I/II/IV MEFA protein protected against a CS6 ETEC strain (B7A) colonization at rabbits’ intestines (26), MecVax is expected to protect against B7A intestinal colonization as well. Nevertheless, additional rabbit immunization and challenge studies to demonstrate that MecVax also protects against intestinal colonization by ETEC strains expressing the other five adhesins (CS1 to CS5) will further validate this vaccine candidate, and surely future clinical efficacy studies will eventually confirm its ETEC vaccine candidacy. Additionally, we need to point out that bacteria intestinal colonization was measured 24 h postinoculation, and future studies with an extended monitoring schedule will be needed to evaluate vaccine lasting protection against ETEC intestinal colonization and perhaps also to monitor shedding of the challenge strains.

Data from the current study documented that intramuscular immunization of protein-based MecVax product protects against ETEC intestinal colonization, the essential step to initiate and to progress ETEC infection. This may expand further investigation of protein-based injectable vaccines against enteric infectious diseases. Parenteral immunization is suggested to enable a vaccine to protect against mucosal diseases (35). Indeed, early studies on cholera vaccine candidates (36, 37) and more recently on other ETEC vaccine candidates (15, 38) showed that parenteral immunization of a protein- or lipopolysaccharide (LPS)-based vaccine candidate induces IgG and/or IgA antibodies to protect against cholera or ETEC diarrhea. While local mucosal immunity, especially that provided by seIgA antibodies, is believed to play a more important role against enteric infections, the current study indicated that rabbits intramuscularly immunized with CFA/I/II/IV MEFA protein or MecVax were protected from ETEC colonization at small intestines, even when no apparent anti-adhesin IgA antibodies were detected. It is worth mentioning that raised OD readings were observed from the wells incubated with cecum content suspensions of the rabbits immunized with MecVax or the adhesin antigen compared to those incubated with control rabbit cecum content samples. This suggested that very low levels of IgA responses may be mounted in the immunized rabbits.

It was reported that low levels of IgA responses were detected in Aotus nancymaae or human volunteers after parenteral administration of ETEC antigens and that a high dose of adjuvant dmLT attributed to elicitation of IgA response (15, 38, 39). Future rabbit immunization studies with MecVax adjuvanted with a higher dose of dmLT should help us to decipher the role played by dmLT in mounting vaccine antigen-specific IgA responses and whether an elevation of IgA response protects rabbits better from ETEC intestinal colonization. Inclusion of dmLT adjuvant (which also induces anti-LT antibodies), however, compromises determination of vaccine-induced anti-LT antibodies for neutralization activity against LT enterotoxicity or protection against LT-mediated clinical diarrhea. Additionally, the current protocol of antibody adherence inhibition assay may need to be optimized, including the use of different cell lines (or rabbit small intestinal cells to be more comparable with *in vivo* rabbit challenge data), since data from this *in vitro* assay significantly underestimated anti-adhesin antibody functional activities against ETEC adherence. *In vitro* antibody adherence inhibition assay showed that H10407 adherence to Caco-2 cells was reduced by 62% to 72% after incubation with the serum from the rabbits immunized with CFA/I/II/IV MEFA protein or MecVax. While these levels of reduction at adherence were significant statistically, they did not mirror the 99.9% reduction of H10407 intestinal colonization in immunized rabbits from the *in vivo* challenge study.

In conclusion, protein-based multivalent ETEC vaccine candidate MecVax, when administered intramuscularly, induced protective antibodies not only against STa or LT toxin-mediated clinical diarrhea (a pig challenge model from a previous study) but also against ETEC intestinal colonization (a rabbit colonization model from the current study). MecVax can become a truly broadly protective vaccine candidate for ETEC diarrhea if future rabbit colonization studies confirm its protection against intestinal colonization from ETEC bacteria expressing the other adhesins. The current study also affirmed that parenteral immunization of a protein-based subunit vaccine can protect against ETEC intestinal colonization, the essential step of ETEC infection, potentially accelerating vaccine development against enteric diseases.

## MATERIALS AND METHODS

### Ethical statement.

Rabbit immunization and challenge complied with the Animal Welfare Act (1996 National Research Council Guidelines), PHS Policy on Humane Care and Use of Laboratory Animals, and the USDA Animal Welfare Act Regulations. The research protocol (no. 21212) was approved by the Institutional Animal Care and Use Committee (IACUC) of University of Illinois at Urbana-Champaign. Animal studies were supervised by institutional attending veterinarian and staff.

### Bacteria strains used in the study.

Recombinant E. coli strains 9471 and 9472 were used to express two MecVax protein antigen components, toxoid fusion 3xSTa_N12S_-mnLT_R192G/L211A_ and CFA/I/II/IV MEFA (17). ETEC H10407 (CFA/I, LT, STa) provided by D. A. Sack at Johns Hopkins University was used as the challenge strain in adult rabbit colonization model study. CS1 or CS2 E. coli recombinant strains THK38/pEu405 and DH5α/pEU588 (provided by J. R. Scott from Emory University) (40, 41), CS3 or CS4/CS6 ETEC isolates E116 and E106 (provided by A. M. Svennerholm from University of Gothenburg), CS5/CS6 isolate UM 75688 (provided by D. A. Sack), and CS6 strain 2423 ETP98066 (a gift from J. M. Fleckenstein at Washington University at St. Louis) (42) were used in antibody adherence inhibition assays.

### Vaccine immunogens and adjuvant used in rabbit immunization.

Three treatments were included in rabbit intramuscular immunization: MecVax (18), at a dose of 200 μg toxoid fusion protein 3xSTa_N12S_-mnLT_R192G/L211A_ (in 200 μL) premixed with 200 μg CFA/I/II/IV MEFA protein (in 200 μL), CFA/I/II/IV MEFA protein (200 μg in 200 μL), and PBS (200 μL) as the control. Holotoxin-structured double mutant heat-labile toxin, dmLT (LT_R192G/L211A_) (43), 1 μg per dose, was included in three treatment groups.

### Rabbit intramuscular immunization.

Twelve New Zealand White (NZW) rabbits of 1.2 to 2.0 kg body weight (Charles River Laboratories, Wilmington, MA), both sexes, were used for immunization. Four rabbits per group were intramuscularly injected with MecVax, CFA/I/II/IV MEFA protein, or PBS, to the epaxial muscles (between the hindlimb and the vertebral column). The injection site was shaved and cleaned with 70% ethanol wipe. Two boosters of the same dose of the primary were followed at an interval of 2 weeks.

Rabbits were observed daily after injection. Blood (via ear vein) and fecal pellets were collected from each rabbit before the primary and 2 weeks after each injection. Fecal pellets were suspended in fecal reconstitution buffer (10 mM Tris, 100 mM NaCl, 0.05% Tween 20, 5 mM sodium azide [pH 7.4]) supplemented with protease inhibitor phenylmethylsulfonyl fluoride (0.2 mg/mL), 1 g fecal pellet in 5 mL buffer (1:6 dilution), and centrifuged (13,000 × *g* for 10 min). Fecal suspension supernatant was collected. Rabbit sera and fecal supernatant samples were stored at −80°C until use.

### Rabbit orogastric inoculation with ETEC strain H10407.

Two weeks after the second booster, all rabbits were orogastrically inoculated with ETEC strain H10407. Rabbits were first administered with antacid famotidine (0.5 to 1.0 mg/kg body weight) 2 to 3 h prior to challenge. Rabbits were sedated with intramuscular injection of dexmedetomidine (0.05 to 0.25 mg/kg body weight) and then anesthetized with 3 to 5% isoflurane in a gas chamber. Each rabbit was then administered with 5 mL sodium bicarbonate, challenged with 1 × 10^11^ CFU H10407 (in 1 mL PBS), and followed with 5 mL sodium bicarbonate, via orogastric intubation. Afterwards, rabbits received atipamezole (0.05 to 0.25 mg/kg body weight) intramuscularly. Rabbits were closely monitored during 24 h postinoculation and recorded for any abnormality. Though diarrhea and dehydration were not expected, we monitored stool formation and conducted skin test by pulling up and releasing skin at scruff area to examine if skin went back quickly.

After 24 h postinoculation, rabbits were euthanized after sedation with dexmedetomidine, anesthesia with 5% isoflurane, exsanguination, and intracardiac injection of KCl. At necropsy, fluid accumulation in intestines and cecum was examined, and cecum contents and distal ilium segment (10 cm) were collected from each rabbit. Cecum contents were suspended in fecal reconstitution buffer, and supernatant was collected after centrifugation. Ileum segment was cut open and rinsed with PBS to remove feces, weighed, and ground in ice-cold PBS (1 g in 9 mL, a 1:10 dilution). Ground ileum tissue solutions were serially diluted, plated on MacConkey agar plates, and cultured overnight at 37°C. CFU were counted and recorded. Five to 10 colonies randomly selected from up to five plates were PCR screened with primers specific to ETEC CFA/I adhesin (CfaB-F, 5′atgaaatttaaaaaaactattggtgcaatg-′3, CfaB-R, 5′-ggatcccaaagtcattacaagagatac-′3).

### Antitoxin and anti-adhesin antibody titration of rabbit serum, fecal, and cecum content.

Serum and fecal samples collected from each rabbit before the primary and 2 weeks after the final booster, as well as cecum content suspension samples collected at necropsy, were titrated in ELISAs for IgG and IgA responses to the seven ETEC adhesins and two enterotoxins targeted by MecVax. As described previously (17–19), heat-extracted fimbrial adhesin CFA/I, CS1, CS2, CS3, CS4, or CS5, recombinant protein CS6 adhesin subunit CssA, or cholera toxin (CT; Sigma) was coated to 2HB plates (100 ng per well), and STa-ovalbumin conjugates were coated on Costar plates (10 ng per well) overnight at 4°C. After blocking with 5% nonfat milk (in 150 μL PBS with Tween 20 [PBST] per well) for 1 h at 37°C, wells were washed with PBST and incubated with 2-fold serial dilutions of rabbit serum, fecal supernatant, or cecum content suspension samples for 1 h at 37°C. This was followed with washes with PBST, incubation with horseradish peroxidase (HRP)-conjugated goat-anti-rabbit IgG or IgA secondary antibodies (1:5,000; Bethyl Laboratories, Montgomery, TX), and then washes with PBST and incubation with 3,3′,5,5′-tetramethylbenzidine (TMB) microwell peroxidase substrate system (2-C; Fisher Scientific). IgG and IgA titers were calculated by multiplying the highest serum dilution giving an OD_650_ of >0.3 (after subtraction with background OD value, typically 0.05 to 0.1) with the adjusted OD; antibody titers were expressed in a log_10_ (IgG) scale as described previously (17, 44–46).

### Rabbit antibody adherence inhibition against *E. coli* or ETEC producing the seven adhesins (CFA/I, CS1 to CS6).

Rabbit serum antibody *in vitro* activity against adherence from E. coli or ETEC bacteria expressing the seven adhesins targeted by MecVax or CFA/I/II/IV MEFA protein was measured in adherence inhibition assays with Caco-2 cells (ATCC no. HT-37). As described previously (17, 24, 44, 46), E. coli recombinant strains expressing CS1 or CS2 or ETEC strains producing CFA/I, CS3, CS4/CS6, CS5/CS6, or CS6 adhesin (1.25 × 10^4^ bacteria), after incubation with 15 μL heat-inactivated rabbit serum sample from the group injected with MecVax, CFA/I/II/IV MEFA protein, or PBS for 30 min at room temperature (50 rpm), were added to 95% to 100% confluent Caco-2 cells (ATCC HTB-37) cultured in 48-well plate wells, at a multiplicity of infection (MOI) of 10 bacteria per cell. After incubation in a 37°C CO_2_ incubator for 1 h, cells were washed with PBS (to remove nonadherent bacteria), dislodged, and harvested. Collected materials were serially diluted, plated on MacConkey agar plates, and cultured 37°C overnight. E. coli or ETEC bacteria (CFU) were counted and recorded. Bacterial adherence was presented in percentage, with bacteria (CFU) adherent to Caco-2 cells treated with the control rabbit serum as 100%; subtraction of bacterial adherence percentage from 100% was referred to as adherence inhibition or reduction.

### Statistical analyses.

Data of antibody titers (in log_10_), antibody adherence inhibition activities (in percentage), and ETEC colonization at small intestines (CFU in log_10_) from different treatment groups were presented as means and standard deviations. Differences between two treatment groups were analyzed using one-way analysis of variance (ANOVA) from GraphPad Prism 7 (San Diego, CA). A *post hoc* (Tukey’s test) was used to calculate *P* value; a *P* value of less than 0.05 indicated significant difference.
